# Isolation Improvement in Reflectarray Antenna-Based FMCW Radar Systems

**DOI:** 10.3390/s22228972

**Published:** 2022-11-19

**Authors:** Hesham Yamani, Jihwan Yoon

**Affiliations:** Electrical and Biomedical Engineering Department, University of Nevada, Reno, 1664 N. Virginia Street, Reno, NV 89557, USA

**Keywords:** RF motion sensor, reflectarray antennas, patch antenna array, passive gain enhancement, numerical modeling

## Abstract

This paper presents an optimization of reflectarray-based RF sensors for detecting UAV and human presence. Our previous human detection radar system adapted a center-fed reflectarray antenna to a commercially available radar system, successfully increasing the gains of the transmit (TX) and receive (RX) antennas by 21.18 dB and the range for detecting human targets 3.4 times. However, because the TX and RX antennas were placed in the focal point of the reflectarray, the TX signal reflected by the reflectarray was directly propagated into the RX antenna, causing desensitization or damage to the receiving circuit if high powers were used. To reduce this direct reflection, we propose a novel radar antenna configuration in which the TX and RX antennas are placed back-to-back with each other. In this configuration, the RX antenna does not directly face the reflectarray, thus direct path between the TX to RX through the reflectarray is removed. The results demonstrate that this approach achieves the optimum isolation level of 51.3 dB. With the reflectarray, the TX antenna gain increases to 30.6 dBi, but the RX antenna gain remains at 16 dBi since the RX antenna does not utilize the reflectarray. The TX and RX gain difference (14.6 dB) is a trade-off for good isolation and may be reduced by utilizing a high-gain receiver amplifier.

## 1. Introduction

Ongoing research aims to develop low-power and affordable perimeter security solutions for military and civilian usage that prevent unauthorized UAV or intruder access, such as critical infrastructure sector (CIS) protection. Microwave radar sensors can detect targets all day and night, perform in less optimal lighting and weather conditions, have reliable long-range detection with little to no false alarms and have very wide area coverage compared to other motion sensors such as image sensors, making it an attractive solution. The recent usage of the radar sensors has shifted from simple target detections to more sophisticated and active applications, such as target classification, pose estimation and autonomous driving with the help of advanced data processing, such as machine learning through wireless communication [1,2]. However, these newly emerging low-power and miniaturized portable radar systems still suffer from the classic problems associated with low-power sensors, such as short-range and low doppler resolution. The research focuses particularly on affordable, high-gain, lightweight, potentially solar- or battery-operated radar systems with low power consumption, since many CISs are built in remote locations where stable power is not guaranteed.

**Figure 1:**
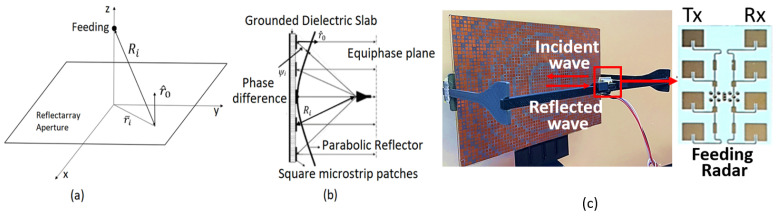
The reflectarray antenna geometry showing (**a**) a 3D view of a feeder in the focus point of the reflectarray aperture, (**b**) a cross-sectional view showing the phase ($\psi_{i}$) required to be adjusted for a constructive phase in direction and (**c**) the final reflectarray geometry with the feeder placed 10 cm away.

Our laboratory has studied methods that improve the range of the radar sensors using low power components or passive hardware for those portable radar systems, as the passive components can help to overcome the disadvantages associated with active components, such as complexity, high cost, reproductivity and thermal constraints [3,4]. We were particularly interested in one method, the reflectarray, as it is passive, lightweight, and capable of significantly boosting antenna gain. The reflectarray is typically used for communication systems but is rarely used in radar systems, which provided an opportunity to investigate the feasibility of the reflectarray as a range-increasing element in our past works [5]. Our research focuses mainly on isolation and low gain issues for newly emerging miniaturized and low-power radar sensors that can be utilized in general applications. In this study, a reflectarray is utilized to increase the range of the detection. Previously, a K-band radar transceiver was placed at the focal point of a custom-made center-fed reflectarray to increase the gain of the antenna (Figure 1). With this configuration, the presence of the reflectarray significantly increases the gains for both TX and RX antennas by 21.2 dB (total gain of 31.9 dBi). This antenna gain increase should result in a 3.4 fold range increase based on the manufacturer’s range estimation equation. The measured range increase was 2.84 fold, 20% lower than the estimation, which could be attributed to the challenge of detecting a relatively small human target with the radar 70 m away. Although the range increase by the reflectarray was within the desired range, an unusually large power leakage from the TX to the RX antennas was observed, which is the major drawback of this configuration. This low isolation increases the noise floor by the zero frequency, degrading the detection sensitivity in FMCW radars or even damaging the receiver circuit [6].

In typical radar systems, the low isolation between the TX and the RX antennas is caused by the signal leakage between the TX and RX, the surface waves, and the radiation pattern ripples that can affect the overall radar performance [7,8]. Several radar techniques were already introduced to achieve high isolation between the TX and RX antennas [7]. The leakage cancellation can be achieved using an adaptive delay filter that achieves about 25 dB cancellation around 900 MHz [9]. Kuo et al. proposed a feed-forward technique with a digitally calibrated isolator, which can be used to reduce the leakages and improve the isolation by 9.3 dB [10]. These methods [9,10] are based on the data processing approach, which does not eliminate the existing high isolation problem but would be useful to adapt for the final radar system. Shin et al. proposed novel fiber-optic links with low distortion characteristics and low propagation loss in a distributed FMCW radar system to keep high isolation between the TX and RX antennas and increase radar sensitivity [11]. The idea is to place the TX and RX antenna far from each other (106.2 m), which resulted in 75 dB isolation. Suh et al. proposed a unique method, adapting a long 75-coaxial cable between TX and RX in the FMCW radar system [12]. However, the two implementations above are not suitable for compact and lightweight radar requirements in this project. Adela et al. inserted electromagnetic bandgap (EBG) structures between the TX and RX antennas, which achieved about 40 dB [13]. Gupta et al. used a defective ground plane to reduce the mutual coupling between antennas [14]. The work demonstrated in [15] utilizes the EBG structures between two adjacent antennas. However, the low isolation issue with the proposed system is mainly due to the reflectarray, and hence those technologies would not increase isolation. The idea of adapting a bandgap structure or defective ground is not desirable in our research, since the TX and RX antennas are placed at a focal point of a reflectarray antenna, and any structure inserted between the TX and RX may cause one or all of the antennas to be out of the focal point. Moreover, inserting an EBG structure would increase the TX and RX board size, reducing the total efficiency by a larger feed blockage. Thus, a novel isolation reduction technique specifically for the reflectarray-based radar system is necessary.

The goal of this project is to improve this low isolation (or high leakage) issue for adapting the reflectarray to radar systems. In this study, the RX and TX antennas are placed back-to-back with each other so that the RX antenna does not face the reflectarray, significantly reducing the leakage from the reflectarray to the RX antenna. This method achieved the averaged measured isolation of 55 dB between the TX and RX antennas in the band of 24–24.25 GHz, which is a 28 dB increment compared to the previous work, while the gain of the system is lower than the previous work [5].

## 2. Materials and Methods

### 2.1. Reflectarray Design

The reflectarray is a flat reflector mimicking a parabolic reflector and has been utilized for radar applications [16]. The design procedure of a reflectarray antenna is described in [7,17] and is briefly described as follows. The reflectarray adjusts the phase of the incident wave in the propagation direction by adjusting the phase of the reflected wave. As shown in Figure 1a, the phase on the surface of the aperture from the feeder can be expressed as the product of $k_{0}$ multiplied by $R_{i}$, where $k_{0}$ is the free space wavenumber and $R_{i}$ is the distance from the center of the incident spherical wavefront to point i on the aperture of the reflectarray antenna. The desired reflection phase Ψ can be realized by adjusting the size of each microstrip square patch on the reflectarray (Figure 1b). For a reflectarray antenna to work, it is required that the reflected fields at the different points on the aperture should have a constructive phase in $r_{0}$, where $r_{0}$ is the unit vector in the direction of the main beam. The desired phase distribution Ψ at the center of each ith patch on the aperture of the reflectarray is obtained from Equation (1) [18,19]

(1) $$\psi_{i}=k_{o}\left(R_{i}-{\vec{r}}_{i}\cdot {\hat{r}}_{o}\right)-2\pi N$$

where $r_{i}$ is the position vector from the center of the reflector to the ith patch. The relationship between the phase of the reflected fields vs. the patch size is obtained by computing the reflection by a test patch antenna embedded inside an infinite array structure. The scattering from this patch embedded in an infinite periodic array is formulated by the Electric Field Integral Equation (EFIE) [20], which is solved using the spectral domain moment method (SDMoM). The resulting equation of the electric field is shown in Equation (2)

(2) $$\vec{E}\left(x,y\right)=\frac{1}{4\pi^{2}}\;\sum_{m=-\infty }^{\infty }\sum_{n=-\infty }^{\infty }\tilde{\bar{\bar{G}}}\left(k_{xm},k_{yn}\right).\;\;\tilde{\vec{J}}\left(k_{xm},k_{yn}\right)e^{jk_{xm}\left(x-x{\;}^{\prime }\right)}e^{jk_{yn}\left(\;y-y{\;}^{\prime }\right)}$$

where $k_{xm}$ and $k_{yn}$ are the discretized wavenumber in the x- and y-directions, respectively; $\tilde{\bar{\bar{G}}}\left(k_{xm},k_{yn}\right)$ is the spectral domain dyadic Green’s function; and ${\tilde{\vec{J}}}_{i}\left(k_{xm},k_{yn}\right)$ is the Fourier transform of the current distribution on the surface of the scattering element [21]. After solving Equation (2) using SDMoM, a lookup table of the reflection coefficient phase vs. the patch size variations was obtained to achieve the required phase at each patch location [7]. The reflectarray was designed and fabricated based on the desired phase distribution obtained from Equation (1) and the lookup table.

**Figure 2:**
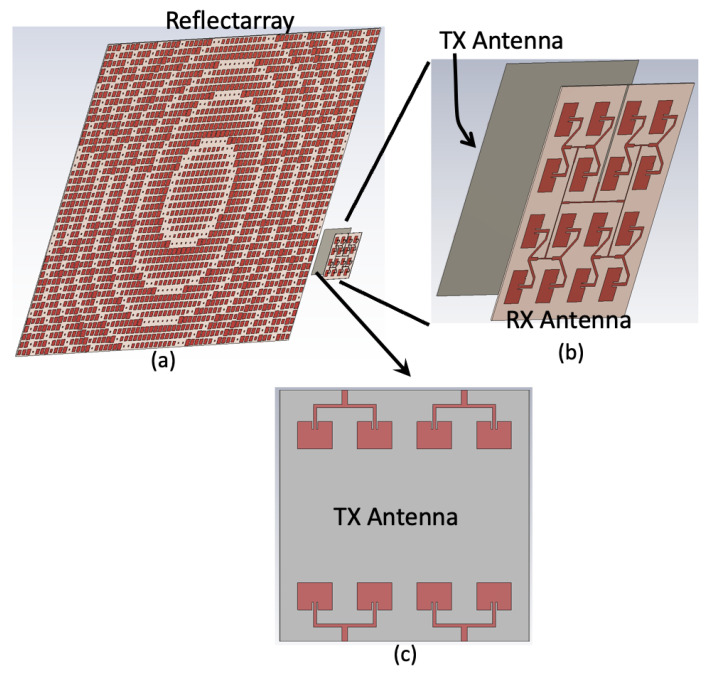
(**a**) The reflectarray fed with the novel TX and RX antenna assembly, where the TX and RX are placed back-to-back. (**b**) The RX antenna board is a 4 × 4 array, and (**c**) TX antenna is four 1 × 2 array antennas facing the reflectarray.

**Figure 3:**
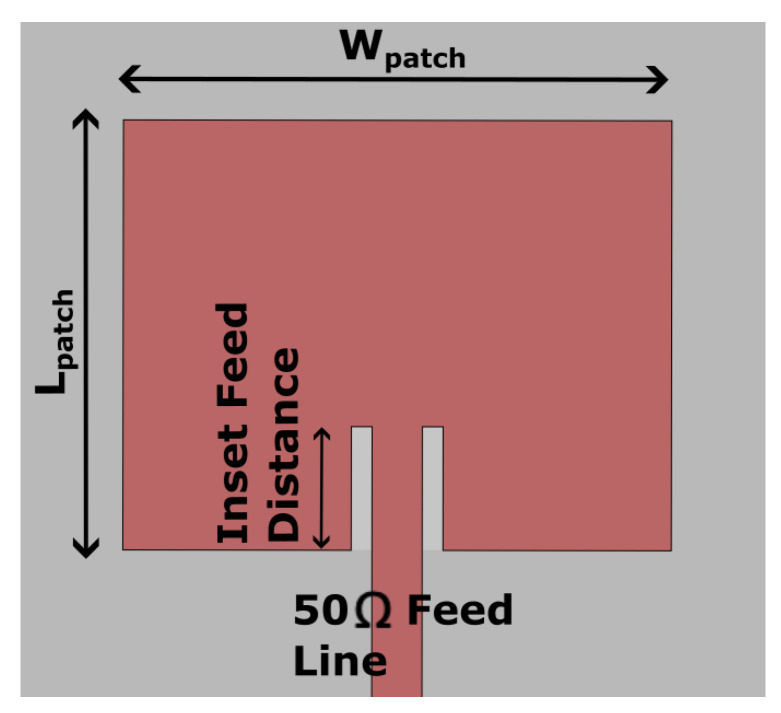
Rectangular microstrip patch antenna with a 50 Ω microstrip feed line.

This reflectarray was designed to reflect the incident signal normal to the surface of the reflectarray, hence a large portion of the reflected signal directly enters the RX antenna, as shown in Figure 1c (red arrows). The result is the low isolation between TX and RX due to this additional leakage, which saturates the receiver amplifiers, causing a loss of sensitivity or damage to the receiver circuits. To overcome the low isolation problem, an antenna assembly is designed explicitly for reflectarray-aided radar applications, in which TX and RX antennas are placed back-to-back to each other to remove the path of the leakage signal going into the RX (Figure 2a,b). Figure 2c shows that the TX antenna board is comprised of four TX antennas, each with a two-antenna patch array. Multiple TX antennas were used in this study to (1) make the TX antenna board similar in size to the RX antenna board (Figure 2c) and (2) utilize commercial radar boards with multiple TX channels, enabling multiple TX antennas to switch or combine antenna radiation patterns for future applications. This switching or constructive beam strategy is in the development stage and out of the scope of this paper, hence it is not discussed further. A wide radiation pattern was implemented for the RX antennas (Figure 2b) to receive echoes by all four transmitted signals. It is anticipated that if the backs of the TX and RX boards are physically touching, the refracted signal from the TX board may enter the RX antenna and cause a lower isolation, so a 0.5 inch (12.75 mm) thick foam material was inserted to separate the TX and the RX antennas (the foam is not shown in Figure 2 for clarity). The TX board was placed 10 cm in front of the same center-fed reflectarray antenna (Figure 2a). As shown in Figure 3a, the design parameter of the conventional microstrip patch includes the length of the patch ($L_{Patch}$ in Figure 2), which is set to nearly half of a wavelength.

### 2.2. TX and RX Patch Antenna Design

The unit cell design for the receiver board is a conventional rectangular microstrip patch antenna, as shown in Figure 3, built on a Rogers 4350B (0.168 mm thick, a dielectric constant of 3.66 and a loss tangent of 0.0037). As shown in Figure 3, the design parameter of the conventional microstrip patch includes the length of the patch ($L_{Patch}$ in Figure 3), which is set to nearly half of a wavelength. The conventional bandwidth (*BW*) was calculated from Equation (3).

(3) $$BW=\frac{\Delta f}{f_{o}}$$

The *BW* (24–24.25 GHz) of the patch antenna is only 1.04 %, satisfying the small *BW* requirement of the FMCW radar system and is the reason why a rectangular patch antenna was used for this application. The width of the patch ($W_{Patch}$ in Figure 3) was calculated from the following Equation (4)

(4) $$W_{Patch}=\frac{1}{2f_{r}\sqrt{\mu_{o}\epsilon_{o}}}\sqrt{\frac{2}{\epsilon_{r}+1}}$$

where $f_{r}$ is the center frequency (24.125 GHz), $\epsilon_{r}$ is the relative permittivity of the substrate material, $\mu_{o}$ is the permeability of the free space and $\epsilon_{o}$ is the permittivity of the free space. Using the substrate thickness (h) and the width of the patch, the increment in the length of the patch can be calculated using Equation (5)

(5) $$\frac{\Delta L}{h}=0.412\frac{\left(\epsilon_{reff}+0.3\right)\left(\frac{W}{h}+0.264\right)}{\left(\epsilon_{reff}-0\cdot 258\right)\left(\frac{W}{h}+0\cdot 8\right)}$$

where the $\epsilon_{\mathrm{reff}}$ is the effective dielectric constant. The physical length of the patch was obtained from the following Equation (6):

(6) $$L_{Patch}=\frac{1}{2f_{r}\sqrt{\epsilon_{reff}}\sqrt{\sqrt{\mu_{o}\epsilon_{o}}}}-2\Delta L$$

The radiation pattern of an array can be obtained by multiplication of the unit cell radiation pattern and array factor [22]. The array factor for a planar antenna array was calculated from the following Equation (7):

(7) $$AF=\sum_{n=1}^{N}I_{1n}\left[\sum_{m=1}^{M}I_{m1}e^{j\left(m-1\right)\left(kd_{x}\sin\;\theta \;\cos\;\varphi +\beta_{x}\right)}\right]\;e^{j\left(n-1\right)\left(kd_{y}\sin\;\theta \;\cos\;\varphi +\beta_{y}\right)}$$

**Figure 4:**
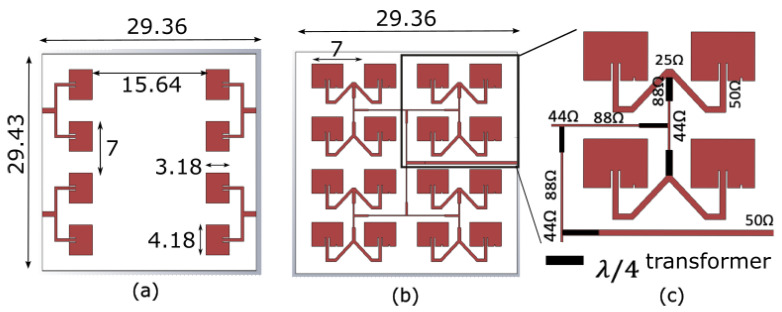
Dimensions of the final (**a**) TX and (**b**) RX antennas optimized in the band of 24–24.25 GHz. (**c**) The upper half of the RX antenna showing detailed design parameters, including the impedances of each line and λ/2 transformers.

Here, $I_{m1}$ and $I_{1n}$ are the excitation coefficients of each antenna element in the x-direction and the y-direction, respectively, and *dx* and *dy* are the distance between the antenna elements in the x-direction and y-direction, respectively, where they are chosen to be λ/2 to avoid any grating lobes. *M* and *N* are the number of antenna elements in the x-direction and the y-direction, respectively. *k* is the wavenumber, $\beta_{x}$ and $\beta_{y}$ are the progressive phase shift between the antenna elements in the *x*-axis and y-direction, respectively. Both $\beta_{x}$ and $\beta_{y}$ are chosen to be zero to have radiation in the broadside direction. θ is the angle measured with respect to the *z*-axis, and ϕ is the angle measured from the *x*-axis. Here, the antenna elements are assumed to be identical and isotropic sources. Figure 4a,b show the resulting location of each element. The feeding network design procedure involves matching the impedance of each antenna to the 50 Ω feeding line using multiple λ/4 impedance transformers. Figure 4a,b show the designed corporate feeding network in the receiver design. Detailed impedance values in the feed network at different locations are shown in Figure 4c, where the input impedance (50 Ω) of each antenna is connected to four of the quarter wavelength transformers on the way to the 50 Ω feed line of the antenna port. It should be noted that the minimum dimension (0.127 mm or 88 Ω) in the circuit results from the minimum line width set by the selected board manufacturer PCB Prime (Aurora, CO, USA). Once the feeding network and antenna were created, the optimization feature of CST Studio Suite (version 2022) was performed to slightly modify the geometry to match the input impedance of the antenna to 50 Ω in the frequency range of interest. The resulting receiver antenna array (Figure 4a,b) has a linear polarization, which is compatible with the polarization of the square patches of the reflectarray antenna.

### 2.3. Simulations and Measurements of Reflection, Isolation and Maximum Gain

**Figure 5:**
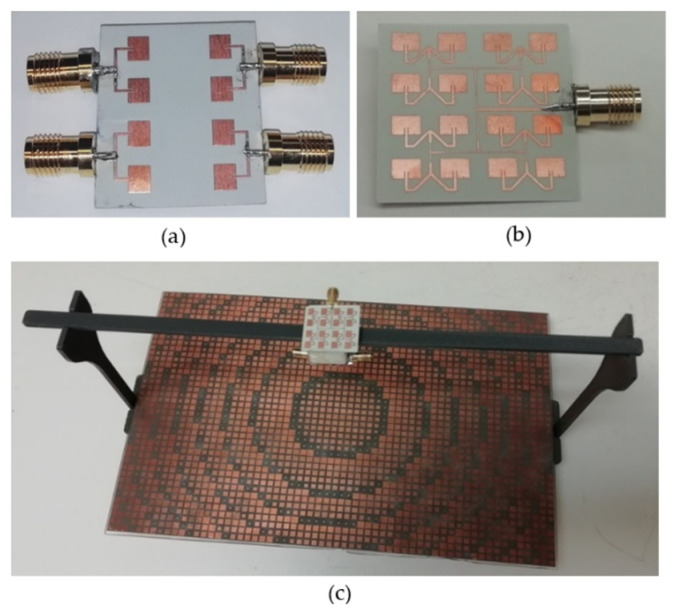
Fabricated (**a**) TX antenna board, (**b**) RX antenna board and (**c**) the reflectarray with the TX and RX antenna assembly.

The designed TX and RX antennas were fabricated, the SMA connectors were soldered to the TX (Figure 5a) and RX (Figure 5b) antennas and their responses were measured using FieldFox RF and Microwave Analyzer (N9962B, Keysight Technologies, Santa Rosa, CA, USA). When measuring responses with the presence of the reflectarray, the antenna assembly was placed 10 cm from the center for the reflectarray (Figure 5c). The isolation levels were obtained by measuring transmission coefficients between the TX and RX antennas without or with the presence of the reflectarray. The same properties were computed using the CST studio for comparison purposes.

### 2.4. Statistical Analysis

In this study, when any measured traces are compared, the traces are expressed in the mean and standard deviations (mean ± S.D.) within the frequency of interest (24–24.25 GHz).

## 3. Results

### 3.1. Reflection Coefficient without the Reflectarray

**Figure 6:**
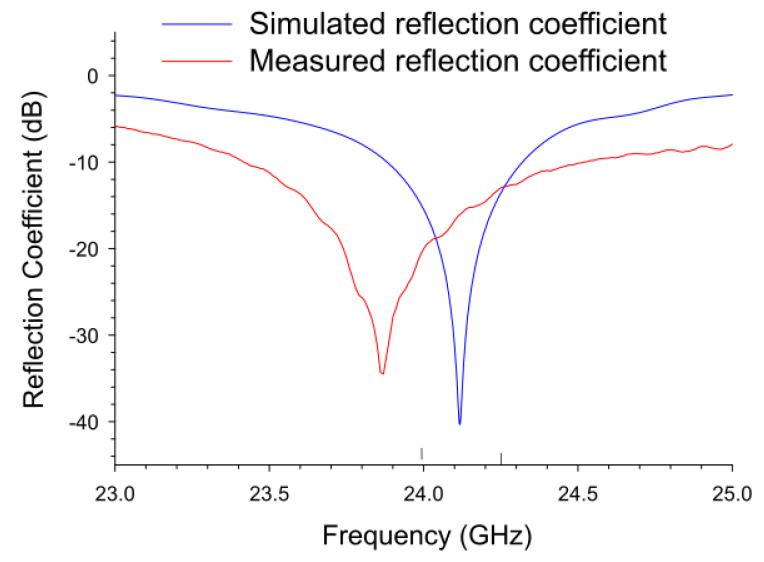
A comparison between simulated and measured reflection coefficients of a representative transmitter antenna.

As shown in Figure 6, the simulated antenna resonated at 24.12 GHz, while the actual resonance was measured at 23.86 GHz, shifting toward the lower frequencies by 0.26 GHz. The reasonable explanation for this shift is a slight difference in the dielectric properties in the actual microstrip board or manufacturing tolerances [23]. The direction of the frequency shift toward lower frequencies indicates the actual dielectric constant is slightly higher than the specification, which aligns with the trend shown in the datasheet where the dielectric constant increases as the frequency increases. Although the measured resonant frequency was not shown within the frequency range of interest, the reflection level in this range was between −20.3 and −13.0 dB, which would not affect the FMCW radar operation and was used as it is.

### 3.2. Isolations without and with the Reflectarray

**Figure 7:**
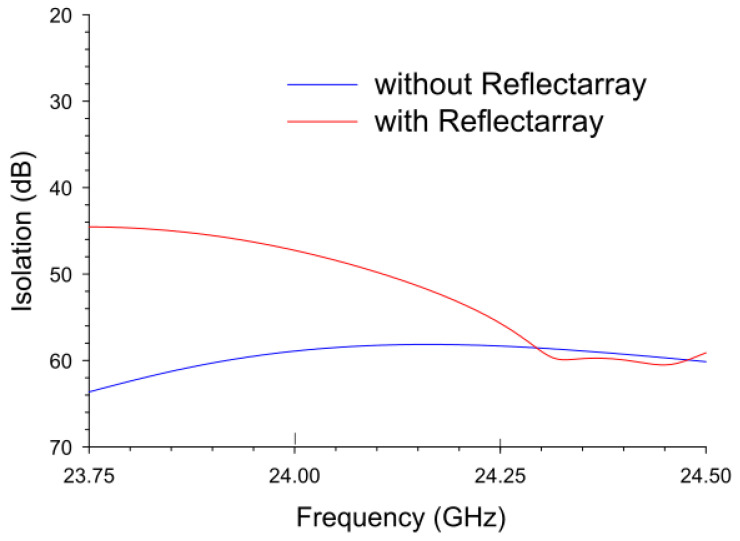
The average of simulated transmission coefficients between the four transmitting antennas and the receiving antenna without and with the presence of the reflectarray antenna.

The simulated and measured isolation levels were obtained using the configuration shown in Figure 2a and Figure 5c. As discussed, the transmitter board is composed of four transmitting antennas, resulting in four isolation traces between the TX antennas and the RX antenna. The results demonstrated that the computed isolations by the four traces were almost identical to each other (data not shown), and for comparison, the four isolation traces without reflectarray were averaged and depicted in Figure 7. Numerical modeling clearly demonstrated that a desired high isolation (58.8 ± 0.21 dB) was obtained in the frequency range of interest.

The high isolation level was anticipated because the radiation patterns of both the transmitters and receiver are placed in opposite directions, causing minimal leakage of power transferring from the TX to RX antennas. Moreover, the averaged isolation in the frequency of interest with this configuration was 31.3 dB higher than the conventional radar antenna case (Figure 1c, inset), where the two antennas are in the same plane and closely located. When the reflectarray was placed 10 cm away (red trace in Figure 7), the isolation was decreased in the lower frequencies, resulting in the average and standard deviation of the isolation level of 51.3 ± 2.41 dB with the highest value of 47.2 dB at 24 GHz.

**Figure 8:**
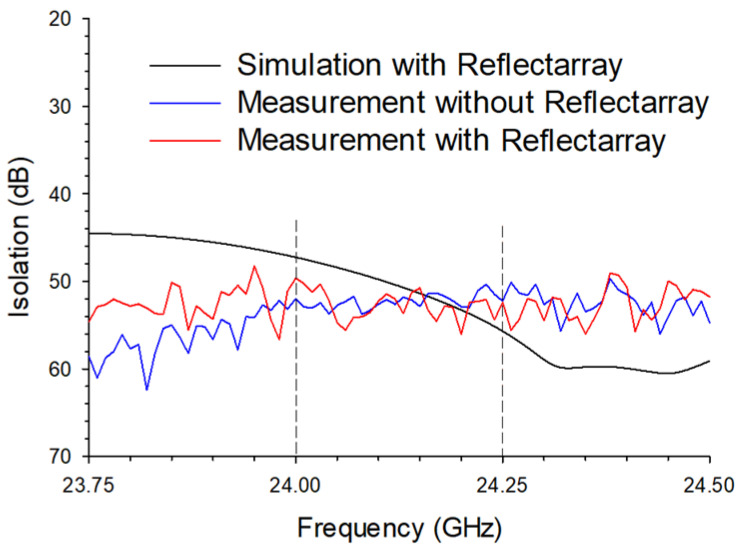
The measured isolations between the transmitting antenna and the receiving antenna without and with the reflectarray antenna. All four isolations were averaged.

This decrease (average decrease of 7.5 dB) was due to the reflected power from the reflectarray that leaked into the receiver antenna via refraction at the edge of the receiver board. Although the reflectarray partially decreased isolation, the TX and RX antennas are still considered highly isolated (minimum 47.2 dB) and satisfy the design requirement of the radar system. The measurements (Figure 8) showed a similar trend, except that the measured isolations without (blue trace) and with (red trace) the reflectarray were closer to each other compared to the simulation results. When comparing the simulated (black trace) and measured (red trace) isolations with the reflectarray case, the average isolations are 50.9 ± 2.4 and 52.7 ± 1.6 dB, respectively, confirming that the simulation and the measurement in the frequency range of interest were similar (average 1.8 dB difference). The numerical modeling and the measurements confirmed that this configuration improves the low isolation problems of the conventional radar antenna configuration similarly to the inset configuration in Figure 1c.

### 3.3. Antenna Gains

**Figure 9:**
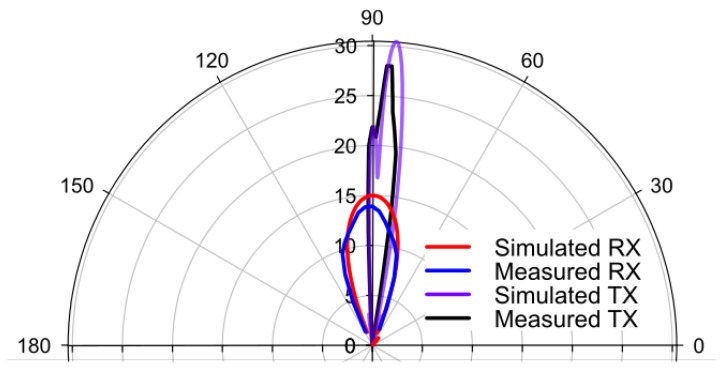
The CST generated and measured gains of the TX antenna 1 and RX antenna for ϕ = 0°.

The numerical modeling and measurements demonstrated that the maximum gain of the transmitter with the presence of the reflectarray was 30.6 and 28 dBi, respectively, as shown in Figure 9, which is similar to the gain obtained by the initial configuration shown in Figure 1c (31.89 dBi), satisfying the gain requirement. The slight decrease is expected because the TX antenna is composed of a two-antenna patch array (Figure 2c) rather than a four-antenna patch array (Figure 1c, inset). The simulated receiver gain was 16 dBi, which is 14.6 dB lower than the transmitter as expected, since the receiver does not use the reflectarray in this configuration. Although the new TX and RX antenna assembly achieved a high isolation level, it suffers from a lower gain for the receiver antenna. However, the high isolation enables the power amplifiers to be inserted at the TX and RX system, overcoming the low receiver gain.

## 4. Discussion

In this study, a novel feeder design for a UAV and human detection system using a reflectarray and FMCW radar was designed and tested. Adapting a standard radar antenna into a center-fed reflectarray system significantly decreases the isolation between the TX and RX antennas since the conventional radar unit faces the reflectarray, causing a large amount of TX signal to flow into the RX antenna. To reduce this leakage by the reflectarray, the TX and RX antennas are placed back-to-back so that the RX antenna faces the opposite direction of the reflectarray. This configuration increases the isolation of the two antennas from 20.04 ± 0.041 to 51.3 ± 2.41 dB with the presence of the reflectarray. The penalty for gaining high isolation is the deduction of 14.6 dB in the RX antenna gain. However, the high isolation enables the radar system to adapt high gain low noise amplifiers for both TX and RX systems, compensating for the low gain of the RX antenna and improving the dynamic range. In Table 1, the isolation level by the back-to-back antenna configuration is compared with other radar isolation improvement methods. Ref. [24] is similar to this application, where the antennas with reflectors were isolated by a metallic serrated wall. Although this method provides a better isolation level, the size of the serrated wall and the two offset parabolic reflectors were much larger than this work. The other two methods [15,25] are designed to increase isolation by inserting either an EBG decoupling structure or a separation wall. Although their antenna configurations are simple and compact, their isolation levels and gains were much lower than this work, attesting that the size and performance of the back-to-back feeder presented in this work are comparable to or better than the existing techniques. In the future, the human detection system with power amplification systems will be implemented and characterized.

## Figures and Tables

**Table 1:** Comparisons of antennas back-to-back method with other methods.

Reference	This Work	[24]	[25]	[15]
Isolation level (dB)	−50 to −55	−62 to −73	−30	−36.7
Gain Tx/Rx dBi	30/16	16/16	6.6/6.6	5.2/5.2
Size (cm)	20 × 30	152 × 51	0.5 × 0.5	21× 29
Frequency (GHz)	24–24.25	24.4–24.8	34.5–35.5	23–25
Technique	Reflectarray Antenna with center-fed back to back	A separation metallic serrated wall	A separation wall	EBG decoupling structure

## Data Availability

Not applicable.

## Abbreviations

The following abbreviations are used in this manuscript:

TX	Transmit
RX	Receive
FMCW	Frequency Modulated Continuous Wave
